# ICTV Virus Taxonomy Profile: Clavaviridae

**DOI:** 10.1099/jgv.0.001295

**Published:** 2019-07-04

**Authors:** David Prangishvili, Tomohiro Mochizuki, Ying Liu, Mart Krupovic, ICTV Report Consortium

**Affiliations:** 1 Department of Microbiology, Institut Pasteur, 25 rue du Dr Roux, 75015 Paris, France; 2 Earth-Life Science Institute, Tokyo Institute of Technology, Tokyo, 152-8550, Japan

**Keywords:** ICTV Report, taxonomy, *Clavaviridae*

## Abstract

The family *Clavaviridae* includes viruses that replicate in hyperthermophilic archaea from the genus *Aeropyrum*. The non-enveloped rigid virions are rod-shaped, with dimensions of about 143×16 nm, and have terminal cap structures, one of which is pointed and carries short fibres, while the other is rounded. The virion displays helical symmetry and is constructed from a single major α-helical protein, which is heavily glycosylated, and several minor capsid proteins. The 5278 bp, circular, double-stranded DNA genome of Aeropyrum pernix bacilliform virus 1 is packed inside the virion as a left-handed superhelix. This is a summary of the International Committee on Taxonomy of Viruses (ICTV) Report on the family *Clavaviridae*, which is available at www.ictv.global/report/clavaviridae.

## Virion

**Fig. 1.**
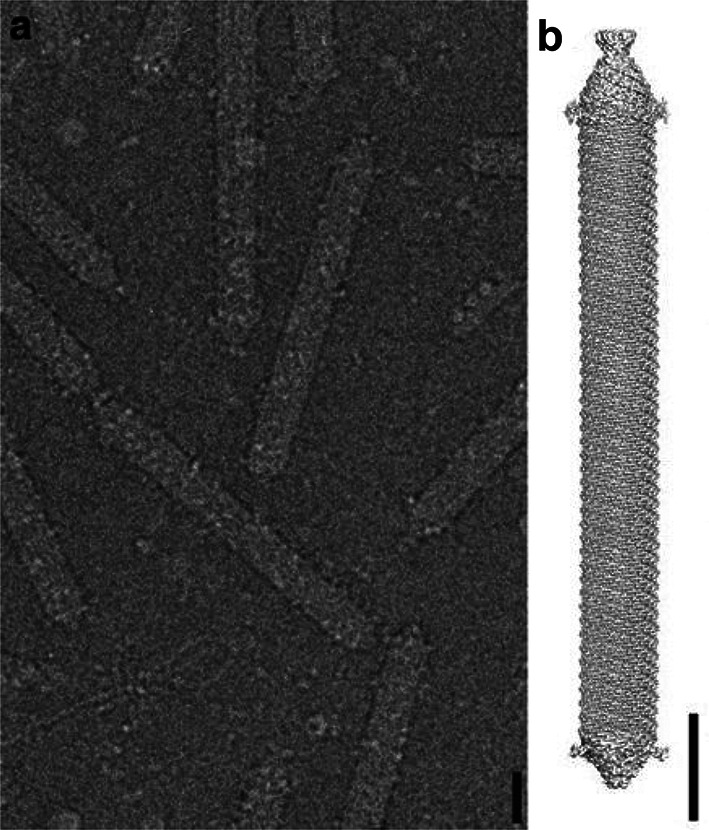
Virions of Aeropyrum pernix bacilliform virus 1. (**a**) Cryo-EM micrograph; (**b**) 3D model shown from the side; scale bars, 10 nm. Reproduced with permission from [2].

Virions of Aeropyrum pernix bacilliform virus 1 are non-enveloped, rigid, rod-shaped particles, with dimensions of about 143×16 nm, terminating with cap structures (Table 1, Fig. 1) [1,2]. The cylindrical virion body has a helical C5 symmetry, whereas the caps display fivefold symmetry [2]. One of the caps is pointed and carries short fibres with five globular protruding domains, whereas the other is rounded and devoid of visible fibres (Fig. 1). The cap structures likely play a role in DNA packaging and host recognition. The virion architecture and manner of DNA packaging are unprecedented among viruses of bacteria and eukaryotes [3,4].

**Table 1.** Characteristics of members of the family *Clavaviridae*

Typical member:	Aeropyrum pernix bacilliform virus 1 (AB537968), species *Aeropyrum pernix bacilliform virus 1*, genus *Clavavirus*
Virion	Rigid bacilliform particles, 143 nm long and 16 nm in diameter, with one end pointed and the other one rounded
Genome	Circular double-stranded DNA of 5278 bp
Replication	Non-lytic, chronic infection
Translation	Not characterized
Host range	Hyperthermophilic archaea from the genus *Aeropyrum*
Taxonomy	Single genus with a single species

Virions are highly thermostable and remain infectious after incubation at 100 °C for 3 h [1]. The high thermostability of the virion is apparently ensured by the structure of the highly glycosylated α-helical major capsid protein (MCP), which is encoded by ORF6-81, and its impeller blade-like arrangement in the virion, in which each MCP molecule makes extensive hydrophobic contacts with six other neighbouring subunits [2]. Positively-charged residues of the MCP interact with the phosphodiester backbone of the double-stranded DNA genome and facilitate its packaging as a left-handed superhelix [2].

## Genome

**Fig. 2.**
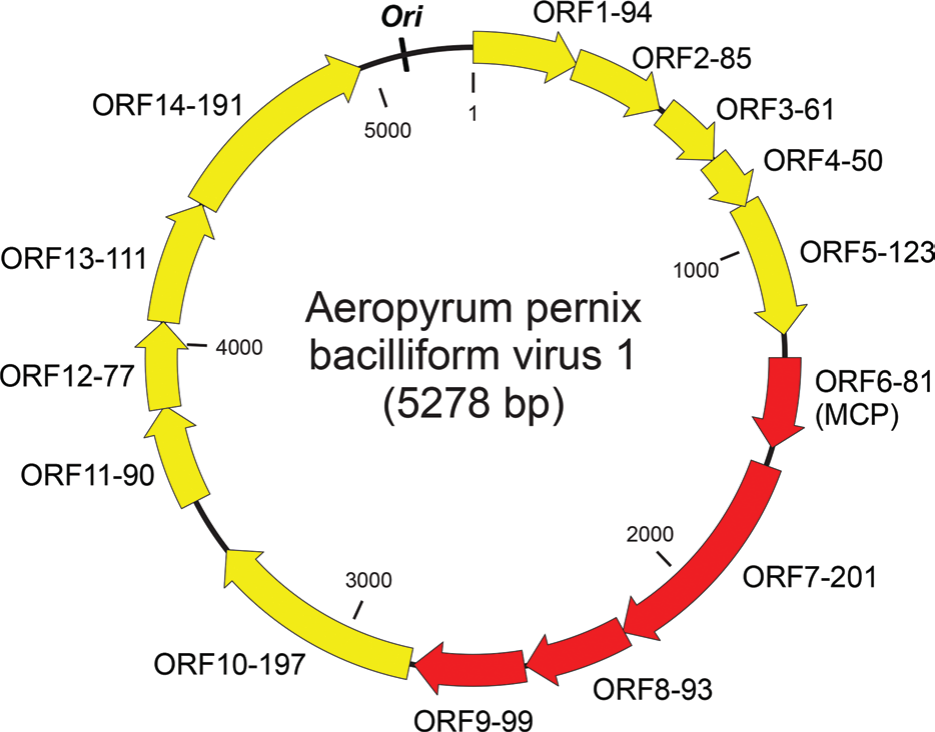
Genome map of Aeropyrum pernix bacilliform virus 1. Each ORF is designated with two numbers; the first number is the order starting from the first ORF after the putative *ori*, and the second reflects the number of amino acids in the encoded protein. Red arrows indicate genes encoding virion proteins. MCP - major capsid protein.

Virions contain a single molecule of circular double-stranded DNA of 5278 bp. The G+C content of the genome is 52.7 %, which is somewhat lower than that for the host chromosome (56.3 %) [1]. The genome contains 14 open reading frames (ORFs) that are larger than 40 codons, all of which are located on the same DNA strand (Fig. 2) [1]. None of the putative gene products share similarity with sequences in public databases [5]. Genes encoding four structural proteins have been identified by protein analysis.

## Replication

The origin of genome replication, *ori*, is most likely localized in the intergenic region between ORF1-94 and ORF14-191 (Fig. 2). Moreover, this region contains a perfect inverted repeat of 19 nucleotides 5′-TGTAGTACACACAATAATT-N_68_-AATTATTGTGTGTACTACA-3′ [1], but the function of the repeat remains unclear. Aeropyrum pernix bacilliform virus 1 virions are released without apparent host cell lysis. The virus does not encode recognizable integrases, which is consistent with the failure to detect integration of the virus genome into the host chromosome. Moreover, the virus does not encode identifiable DNA and RNA polymerases and is likely to depend on the host machineries for genome replication and transcription.

## Taxonomy

The family *Clavaviridae* comprises a single genus *Clavavirus*, with one species, *Aeropyrum pernix bacilliform virus 1*. Virus of this species infects *Aeropyrum pernix*, a member of the hyperthermophilic archaeal order Desulfurococcales within the phylum Crenarchaeota, that grows optimally at 90–95 °C [1]. No relationships with other viruses have been revealed based on structural or sequence similarities.

## Resources

Full ICTV Report on the family *Clavaviridae*: www.ictv.global/report/clavaviridae.
